# LAG-3 palmitoylation-inducing dysfunction of decidual CD4^+^T cells is associated with recurrent pregnancy loss

**DOI:** 10.1186/s10020-025-01361-9

**Published:** 2025-09-29

**Authors:** Liyuan Cui, Fengrun Sun, Xinhang Meng, Yujie Luo, Jinfeng Qian, Songcun Wang

**Affiliations:** Laboratory for Reproductive Immunology, Hospital of Obstetrics and Gynecology, Fudan University Shanghai Medical College, Shanghai, 200090 P.R. China 128 Shenyang Road,

**Keywords:** LAG-3, Decidual CD4^+^T cells, Palmitoylation, Pregnancy, Recurrent pregnancy loss

## Abstract

Recurrent pregnancy loss (RPL) profoundly impacts not only the physical health but also the psychological well-being of women. Despite its profound effects, the underlying pathophysiological mechanisms of RPL remain largely elusive, with few discernible warning signs. Lymphocyte activation gene-3 (LAG-3) is a crucial immune checkpoint that modulates immune responses during infection and tumor. In the present study, we examined the expression of LAG-3 on CD4^+^T cells during pregnancy via cytometry by time-of-flight and flow cytometry. Our findings revealed a higher frequency of LAG-3^+^ decidual CD4^+^T (dCD4^+^T) cells in response to trophoblasts during normal pregnancy. This specific LAG-3^+^ subset of dCD4^+^T cells was found to produce a greater number of anti-inflammatory cytokines. Notably, blocking LAG-3 was highly effective in inhibiting the production of anti-inflammatory cytokines, which is detrimental to the maintenance of pregnancy. A decrease in the number of LAG-3^+^dCD4^+^T cells was correlated with miscarriage. Interestingly, the RNA level of LAG-3 (data analyzed from the two published single-cell databases) remained stable in RPL. Palmitoylation might play a role in regulating LAG-3 expression during RPL, as the palmitoylation of LAG-3^+^dCD4^+^T cells was increased in RPL. Additionally, the general palmitoylation inhibitor 2-bromopalmitate was found to upregulate LAG-3 expression on dCD4^+^T cells both *in vitro* and *in vivo.* Collectively, these findings highlighted the significant roles of LAG-3 in regulating the function of dCD4^+^T cell and maintaining normal pregnancy. Furthermore, they suggested that lower LAG-3 expression on dCD4^+^T cells could serve as a potential biomarker for diagnosis of RPL.

**Supplementary Information:**

The online version contains supplementary material available at 10.1186/s10020-025-01361-9.

## Background

Recurrent pregnancy loss (RPL), which generally refers to the spontaneous loss of two or more pregnancies before the fetus reaching viability(Regan et al. 2023), has a profound impact on the physical and psychological health of women. Although parental or embryonic chromosomal anomalies, uterine anatomic defects, endocrine disorders, infectious factors, and antiphospholipid syndrome are recognized as common culprits behind RPL, a comprehensive diagnostic workup is able to pinpoint the etiology in less than half of the affected couples(El Hachem et al. 2017). The fetus carries both maternal and paternal DNA material, thus pregnancy inherently involves physiological exposure, and often repeated exposure, to genetically foreign antigens of the developing fetus. Pregnant women are immunologically cognizant of these foreign fetal alloantigens, yet they do not mount a rejection response. Consequently, during pregnancy, the maternal immune system is activated to safeguard against infections and to prevent the rejection of the fetus(Arck and Hecher 2013). Maternal-fetal immune dysregulation has been hypothesized as a potential underlying factor in unexplained cases(Yu et al. 2023).

Checkpoints are immune molecules that orchestrate co-stimulatory and co-inhibitory signals, playing a pivotal role in maintaining the delicate immunological balance between tolerance and autoimmunity. Co-stimulatory signals, mediated by activating receptors, can boost the proliferation and cytotoxicity of effector cells, while co-inhibitory signals, transmitted by inhibitory receptors, can curb the proliferation and effector functions of immune cells. Consequently, the expression of inhibitory receptors is essential for fostering appropriate self-tolerance and for sustaining immune homeostasis; however, their overexpression may lead to a diminished capacity of effector cells to mount effective immune responses against pathogens or tumors(Joller et al. 2024). This critical function in regulating immune responses has positioned inhibitory receptors, such as programmed cell death protein 1 (PD-1), cytotoxic T-lymphocyte antigen-4 (CTLA-4), T cell immunoglobulin and mucin domain containing-3 (TIM-3), lymphocyte activation gene-3 (LAG-3), and others, as promising targets for immunotherapy, particularly in the setting of cancer treatment(Kozlowski et al. 2022; Shi et al. 2018).

Although immune checkpoint inhibition has achieved notable success in cancer treatment, its application in patients with underlying autoimmunity is deemed hazardous. Studies have demonstrated that blockade or deletion of LAG-3 in autoimmune-prone contexts or in disease-induced models can lead to a worsening of the condition(Jones et al. 2022). Furthermore, immune checkpoints are engaged from the earliest stages of implantation, serving to avert rejection responses to fetal antigens by the maternal immune system(Phoswa et al. 2023). Notably, memory CD8^+^ T cells, which express high levels of LAG-3, are particularly susceptible to functional exhaustion upon re-exposure to fetal antigen following a previous pregnancy(Kinder et al. 2020). Additionally, LAG-3^+^ regulatory T cells (Tregs) are reported to stimulate maternal tolerance towards the fetus by inhibiting the proliferation of effector T cells(Zhang and Sun 2020). Interestingly, CD4^+^ and CD8^+^ T cells exhibited reduced expression of LAG-3 in early-onset pre-eclampsia when compared to normotensive pregnancies(Liang et al. 2022). Furthermore, the targeted functional neutralization of PD-1/LAG-3 signaling pathways has been associated with near-complete fetal wastage(Kinder et al. 2020). These observations underscore the pivotal role of inhibitory receptors in the preservation of pregnancy.

Regrettably, empirical evidence supporting the immunomodulatory function of LAG-3 in the context of RPL remains scarce. In this study, we aim to bridge this knowledge gap by investigating the regulation of LAG-3 expression in RPL patients. We utilized cytometry by time-of-flight (CyTOF) to explore the source of LAG-3 expression discrepancies in RPL. Additionally, we conducted a series of *in vitro* and *in vivo* functional experiments to elucidate the consequences of these expression changes on the pathogenesis of RPL.

## Materials and methods

### Human samples

This study was approved by the Human Research Ethics Committee of the Obstetrics and Gynecology Hospital, Fudan University. Written informed consent was obtained from each participant. We collected samples from human first-trimester pregnancies, which included those from clinically normal pregnancies (NP) that were terminated for non-medical reasons (*N* = 55, comprising whole peripheral blood, villous and decidual tissues) and those from miscarriages (diagnosed as recurrent pregnancy loss, RPL, with exclusions for cases resulting from chromosomal defects, infection, genetic abnormalities, endocrine issues, anatomic factors, etc., *N* = 26, comprising whole peripheral blood and decidual tissues). The clinical characteristics of the enrolled subjects are summarized in Supplementary Table 1. Immediately upon collection, samples were processed for the isolation of peripheral blood mononuclear cells (PBMCs), trophoblasts, and decidual immune cells.

### Human cell isolation and treatments

PBMCs were extracted from peripheral blood of women with NP or RPL using Ficoll density gradient centrifugation (Huajing, China). Trophoblasts were carefully isolated from the normal villous tissues by trypsin-DNase I (150 U/mL, Applichem, Germany) digestion followed by discontinuous Percoll gradient centrifugation (GE Healthcare, U.S.A.). Decidual immune cells were obtained from the normal or RPL decidual tissue by enzymatic digestion in RPMI 1640 (HyClone, U.S.A.), which was enriched with collagenase type IV (1.0 mg/mL, CLS-1, Worthington Biomedical, U.S.A.) and DNase I (150 U/mL, Applichem, Germany) as described previously(Li et al. 2021). Isolation of CD4^+^ T cells was achieved by magnetic affinity cell sorting using CD4 microbeads (MiltenyiBiotec, Germany).

Freshly isolated trophoblasts were cultured at a density of 2 × 10^5^ cells/mL per well in Matrigel-coated 24-well plates and incubated overnight. The following day, the cells were gently rinsed with phosphate buffer solution (PBS) (HyClone, U.S.A.) to remove any debris. Subsequently, an equal count of PBMCs were added to each well. After co-culture, the immune cells were then harvested and prepared for subsequent flow cytometry analysis to evaluate their phenotypic and functional characteristics.

In certain experimental setups, decidual CD4^+^T (dCD4^+^T) cells were cultured (5** × **10^5^ per well) in the presence of anti-LAG-3 (10 µg/mL, clone L3D10, BioLegend, U.S.A.). To stimulate the cells, Phorbol 12-myrstate 13-acetate (PMA, 50 ng/mL), ionomycin (1 µg/mL) and brefeldin A (10 mg/mL) was added 4 h prior to the conclusion of the culture. After treatment mentioned above, the cells were then collected and subjected to flow cytometry for further analysis.

### CyTOF

To accurately distinguish a broad range of immune cells, we designed a comprehensive panel consisting of 35 antibodies, which were sourced in their purified form from Biolegend (San Diego, United States), BD (San Diego, United States) and Merk (Darmstadt, Germany), The detailed List of antibodies is presented in Supplementary Table 2. These antibodies were conjugated to heavy metals in-house using the Maxpar X8 Multimetal Labeling Kit (Fluidigm, United States) following the manufacturer’s instructions. After initial staining with cisplatin-195Pt (Fluidigm, 201064), cell samples were incubated with cell surface antibodies on ice for 30 min. Subsequently, the samples were permeabilized and stained with intracellular antibodies for 30 min at a temperature of 4 ℃. After washed twice with cell staining Buffer, the antibody-labeled samples were incubated overnight in a solution containing 0.125 nM intercalator-Ir (Fluidigm, United States), diluted in PBS (Sigma-Aldrich, United States) containing 1.6% formaldehyde at a temperature of 4 °C. Prior to analysis, the samples were washed twice, first with deionized water and then with cell acquisition solution, and finally re-suspended at a concentration of 1 × 10^6^ cells/mL in cell acquisition solution containing a 1:20 dilution of EQ Four Element Beads (Fluidigm, United States). The samples were subsequently analyzed using CyTOF2 mass cytometry (Fluidigm, United States) to obtain a high-dimensional profile of the cellular populations.

Data from the CyTOF2 system were collected as.fcs files, a standard format for flow cytometry data. The incorporation of EQ Four Element Beads facilitated the implementation of an R-based normalization technique. Further analysis was performed using FlowSom, an automated dimensionality reduction algorithm integrated into the R software environment.

### Mice

CBA/J female, DBA/2 male, and BALB/c male mice were purchased from Huafukang (Beijing, China). The care and use of these animals adhered to the National Guidelines for Animal Care and Use in Research (China), with all experimental methods being conducted in compliance with these approved guidelines. Eight-week-old CBA/J female mice were mated to BALB/c male mice to establish normal pregnancy models or with DBA/2 male mice to create abortion-prone models. The day of presence of a mating plug was designated as day 0.5 of pregnancy. In certain experiments, pregnant female mice were injected with anti-LAG-3 antibody (clone C9B7W, BioLegend, U.S.A.), or isotype IgG via intraperitoneal(i.p.) at doses of 500, 250, and 250 µg on days 4.5, 6.5, and 8.5 of gestation, respectively, following protocols established in our previous publications(Li et al. 2021). In other experiments, pregnant female mice received intraperitoneal injections of 2-bromopalmitate (2BP) at a daily dose of 32 mg/kg, three times a week. All pregnant mice were monitored and assessed on day 13.5 of pregnancy.

### Preparation of mouse cells

Uteri from pregnant mice were dissected free from the mesometrium, and the fetal and placental tissues were carefully removed from the uterus. The dissected uteri were finely minced and then subjected to enzymatic digestion in RPMI 1640 medium (HyClone, U.S.A.), which was enriched with collagenase type IV (1.0 mg/mL, Worthington Biomedical, U.S.A.) and DNase I (150 U/mL, Applichem, Germany). This digestion process was carried out for 30 min at 37 °C with gentle agitation. Isolated cells were cultured in RPMI 1640, which was supplemented with 10% fetal bovine serum (FBS), 100 U/mL penicillin, 100 µg/mL streptomycin, and 1 µg/mL amphotericin B at 37 °C in a 5% CO_2_ environment for 2 h to remove adherent stromal cells. Then the cell suspensions were collected and treated with anti-CD3 (5 µg/mL, BioLegend, U.S.A.), anti-CD28 (1 µg/mL, BioLegend, U.S.A.), PMA (50 ng/mL, BioLegend, U.S.A.), ionomycin (1 µg/mL, Biolegend, U.S.A.) and brefeldin A (10 mg/mL, BioLegend, U.S.A.) to facilitate intracellular cytokine analysis.

### Single-cell transcriptomics analysis

Single-cell sequencing data were downloaded from the Gene Expression Omnibus (GEO, https://www.ncbi.nlm.nih.gov/geo/) database. The specific datasets used in this analysis were is GSE164449 and GSE130560 (Chen et al. 2021; Wang et al. 2021), both of which consist of single-cell raw data derived from the decidua of three healthy controls and three patients with RPL in the GEO database.

The gene expression matrix was analyzed using the R package Seurat. To exclude red cells and low-quality cells from our analysis, we first filtered out cells with hemoglobin genes (HB_genes) and assessed the quality of the cells using metrics such as nCount (nCount < quantile 0.97 and > 1000), nFeature (nFeature > 300 and < 4000) and high mitochondrial content (> 10%). Once the samples were merged, the expression matrix underwent normalization, and highly variable genes were identified by FindVariableFeatures () function. Principal component analysis was then performed using these highly variable genes, and sample integration was conducted using the Harmony R package. Clustering was completed using FindNeighbors () and FindClusters () functions. To label the cell clusters, we referred to a set of classic marker genes, as indicated in the literature, to annotate each cell type.

### Flow cytometry

Cell surface molecular expression and the production of intracellular cytokines were evaluated using flow cytometry. FITC-conjugated anti-mouse IL-4, IL-10, PE/Dazzle™ 594-conjugated anti-human CD14 or anti-mouse IFN-γ, AlexaeFluor^®^ 647-conjugated anti-human LAG-3 or anti-mouse LAG-3, PE-conjugated anti-human IL-4, cleaved caspase-3, PE/CY7-conjugated anti-human CD56, TNF-α, IL-10 or anti-mouse TNF-α, APC/Fire™ 750-conjugated anti-human CD8, Brilliant Violet 421-conjugated anti-human Ki-67, TGF-β1, Brilliant Violet 510-conjugated anti-human IFN-γ, anti-mouse CD4, Brilliant Violet 605-conjugated anti-human CD4, antibodies (Biolegend, U.S.A.) were used. For intracellular staining, cells were fixed and permeabilized using the Fix/Perm kit (Biolegend, U.S.A.). Flow cytometry analysis was performed on a Beckman-Coulter CyAn ADP cytometer and the data obtained were subsequently analyzed with FlowJo software (Tree Star, U.S.A.).

### Western blot (WB)

The isolated cells were lysed with cold radio-immunoprecipitation (RIPA) buffer (Beyotime Biotechnology, China) supplemented with a protein inhibitor cocktail (MCE, China). Protein concentrations were measured by the BCA Kit. Proteins were separated with SDS-polyacrylamide gel electrophoresis, and then were transferred onto 0.2 μm PVDF membranes (Amersham, Germany). The membranes incubated overnight at 4 ℃ with the primary antibodies (anti-LAG-3 (ab40465, Abcam, U.S.A), anti-Actin (ACTB, ab179467, Abcam, U.S.A)) after blocked with 5% nonfat milk, followed by HRP-conjugated secondary antibody (Jackson, U.S.A) incubation at room temperature for 1 h. At last, the antibody-labeled proteins were detected with chemiluminescence (Millipore, U.S.A) in an Amersham™ Imager 600 (GE Healthcare, U.S.A).

### Click-iT identification of LAG-3 palmitoylation

Cells were treated with 100 µM of Click-iT palmitic acid-azide with gentle mixing, then incubated at 37 °C and under 5% CO_2_ for 6 h. After incubation, the medium was removed, and the cells were washed three times with PBS. Following the washes, lysis buffer (1% sodium dodecyl sulfate in 50 mM Tris-HCl, pH 8.0) containing protease and phosphatase inhibitors was introduced to the cells to facilitate cell lysis. The cell lysate was then centrifuged at 13,000 g at 4 °C for 15 min, and then we transferred the supernatant to a fresh tube and determined the protein concentration using the BCA Protein Assay Kit (catalogue number WB6501; NCM Biotech). Thus, the protein sample was processed with the Click-iT Protein Reaction Buffer Kit (catalogue number C10276; Thermo Fisher Scientific) according to the manufacturer’s instructions. Finally, the biotin- alkyne-azide-plamitic-protein complex was pulled down by streptavidin and were subjected to immunoblotting detection for LAG-3.

### Statistical analysis

Data are tested for normal distribution (Kolmogorov-Smirnov), defining whether the results should be analyzed parametrically or non-parametrically. For the normally distributed data, significance of differences between two groups was determined by Student’s t-test. For the non-normally distributed data, significance of differences between two groups was determined by Mann-Whitney-test. Multiple groups were analyzed by one-way ANOVA with the post-hoc Dunnett t-test using Prism Version 8 software (GraphPad, San Diego, CA, USA). Variables were presented as means and standard error of mean (SEM). For all statistical tests, p- values < 0.05 were considered statistically significant.

## Results

### Comparative expression analysis of LAG-3 in clinically normal first-trimester pregnancies and RPL patients

**Fig. 1:**
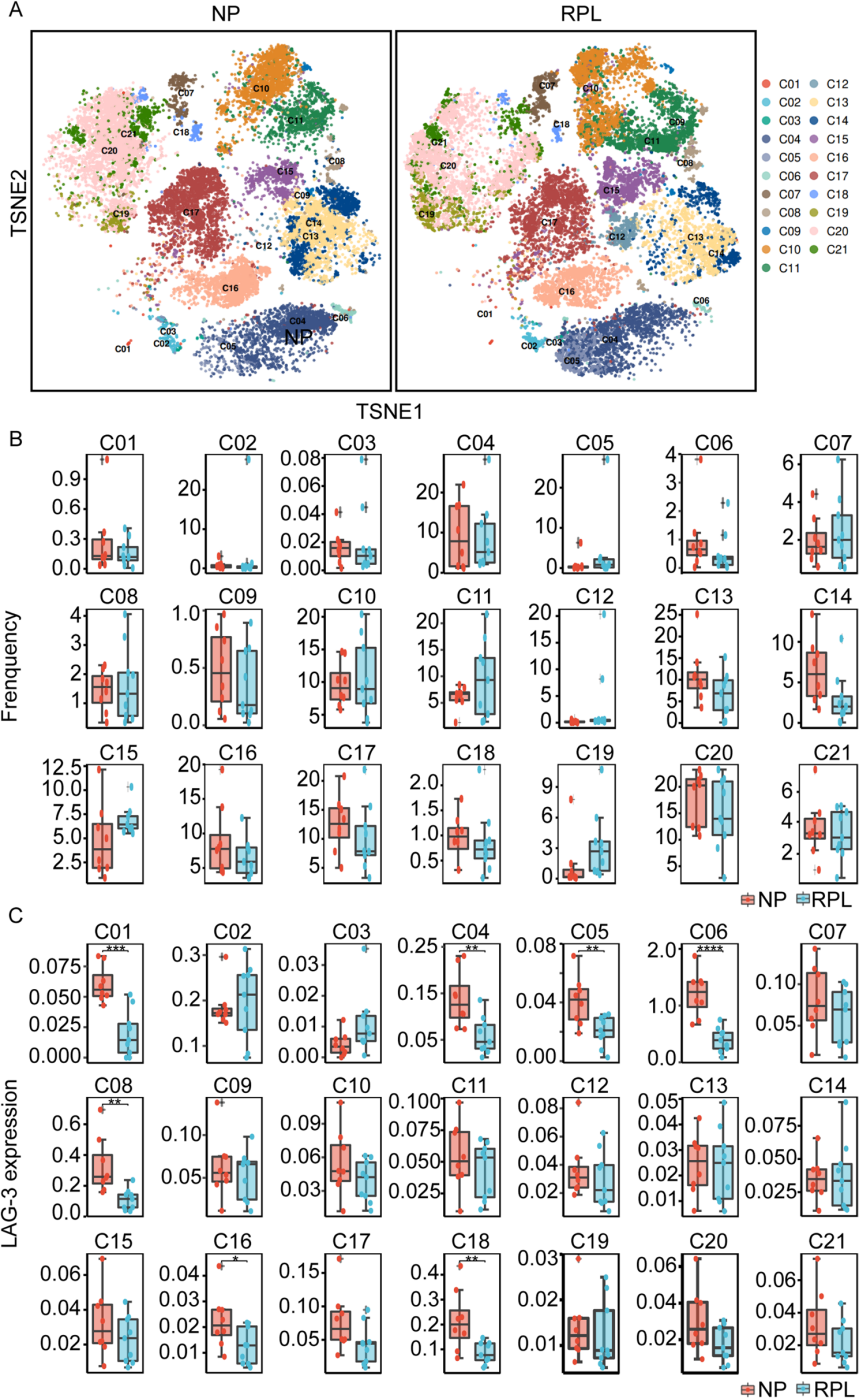
Comparative LAG-3 expression in PBMCs during clinically normal first trimester pregnancies and RPL using CyTOF.A t-distributed stochastic neighbor embedding (TSNE) plot of CyTOF data to show 21 distinct cellular clusters within PBMCs obtained from women with clinically normal first trimester pregnancies (NP, n = 8) and those with RPL (n = 9). B The proportion of cells in different clusters in PBMCs from clinically normal first trimester pregnancies and RPL patients. C Box plots illustrated the normalized LAG-3 expression levels across the identified clusters of PBMCs from clinically normal first trimester pregnancies and RPL patients. *P < 0.05, **P < 0.01, ***P < 0.001, ****P < 0.0001

A comprehensive CyTOF analysis utilizing a panel of 35 metal isotope-tagged monoclonal antibodies was conducted for a global overview of PBMCs from women with clinically normal first trimester pregnancies (normal pregnancy) and those with RPL. According to the similarity of cell surface markers signal, the FlowSOM (Van Gassen et al. 2015) analysis were applied to identify 21 distinct cell clusters (Fig. 1A and S1A-1B). The immune cell subsets, including innate lymphoid cells, neutrophils, T cells, NK cells, B cells, and monocytes, were found to be relative equally represented in both groups (Fig. 1B). The expression of LAG-3 across the entire PBMC population was evaluated (Fig.S1C), revealing no significant difference between normal pregnancy and RPL patients, despite a noticeable decreasing trend in LAG-3 expression in RPL group (Fig.S1D). However, statistically significant difference in LAG-3 expression were observed (Fig. 1C) in cluster 01 (innate lymphoid cells), clusters 04–06 (neutrophils), cluster 08 (CD8^+^T cells), cluster 16 (B cells), and cluster 18 (CD4^+^T cells). These findings suggested that LAG-3 might modulate the function of these cells during RPL.

**Fig. 2:**
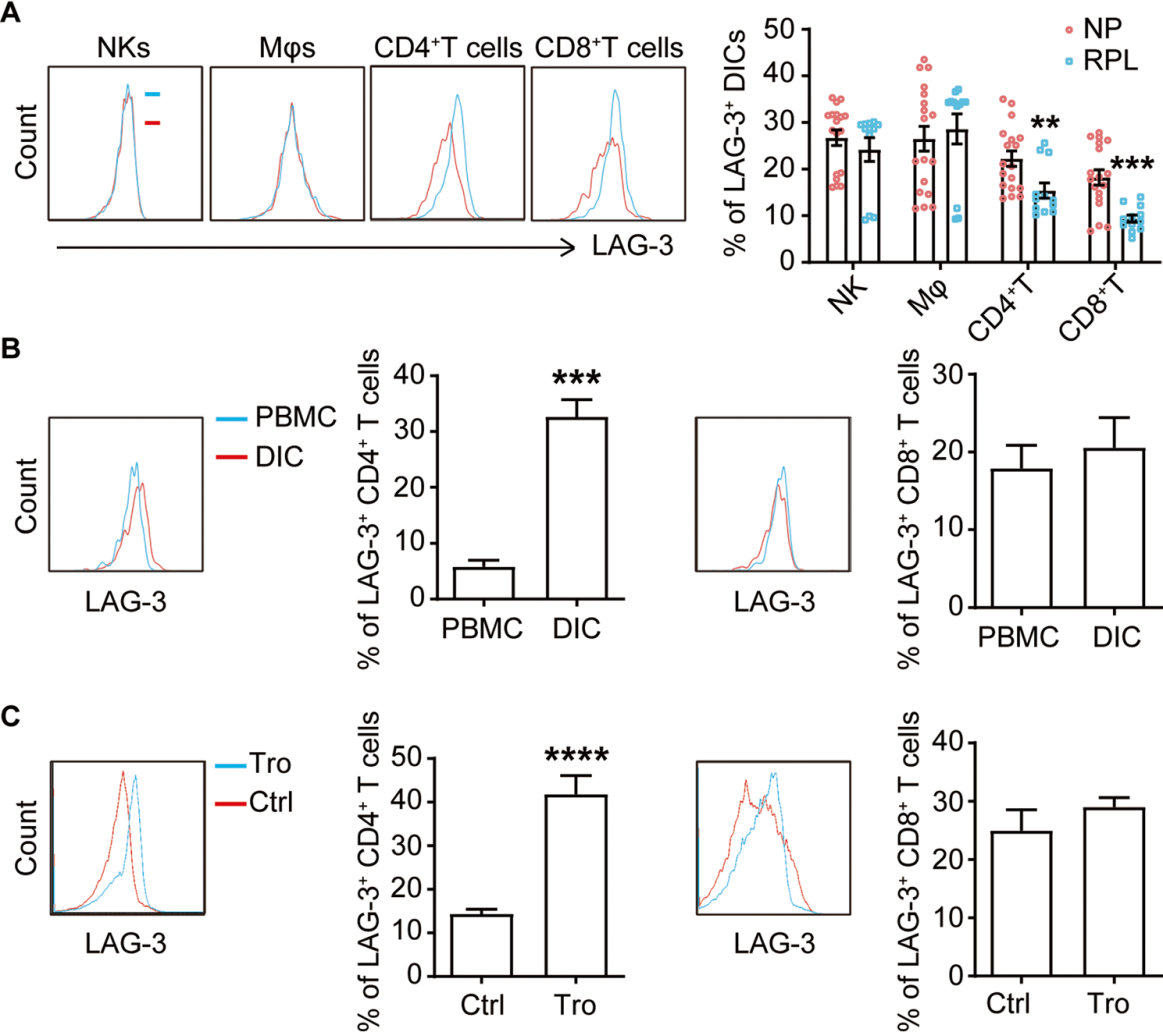
LAG-3 expression on CD4^+^ T cells during human early pregnancy. **A** Flow cytometric analysis (left) and quantification (right) of LAG-3 expression on gated decidual NK cells, macrophages (Mφs), CD4^+^T cells and CD8^+^T cells from decidual immune cells (DICs) during human normal first trimester pregnancies (*n* = 18) and RPL patients (*n* = 12). **B** Quantification of LAG-3 expression on gated CD4^+^T and CD8^+^T cells derived from paired PBMCs and DICs, as measured by flow cytometry (*n* = 9). **C** Quantification of LAG-3 expression on pCD4^+^ T and pCD8^+^ T cells cultured in isolation or in co-cultured with equal numbers of trophoblasts (Tros, *n* = 9). The flow cytometry plots were representative of three independent experiments. Data represented the mean ± SEM, **P < 0.01, ***P < 0.001, ****P < 0.0001

To delve deeper into the role of LAG-3 in maternal-fetal tolerance, we analyzed its expression on major decidual immune cell types (Yang et al. 2019). As shown in Fig. 2A, dCD4^+^T cells and dCD8^+^T cells from RPL patients exhibited significantly lower LAG-3 expression (about 15.39% and 9.369%, respectively) compared to those from normal pregnancies (about 22.25% and 18.22%, respectively). In contrast, the number of LAG-3^+^NK cells and LAG-3^+^ macrophages were comparable between the two groups. We further compared the expression levels of LAG-3 on CD4^+^T cells and CD8^+^T cells isolated from paired decidua and peripheral blood from normal pregnancies. LAG-3 was significantly more abundant on dCD4^+^T cells than on peripheral CD4^+^T (pCD4^+^T) cells. Conversely, there was no difference in LAG-3 expression between dCD8^+^T and pCD8^+^T cells (Fig. 2B).

In placental mammals, trophoblasts invade the decidua during a successful pregnancy, guiding decidual immune cells to adopt a regulatory phenotype essential for maternal-fetal tolerance(Du et al. 2014). In the co-culture system of trophoblasts, trophoblasts significantly enhanced LAG-3 expression on CD4^+^T cells, but had no effect on CD8^+^T cells (Fig. 2C). These observations implied that LAG-3 signaling might participate in the pathogenesis of RPL by regulating CD4^+^T cell functions. Consequently, we selected CD4^+^T cells as the focus for subsequent experimental investigations. And the higher LAG-3 expression on dCD4^+^T cells of normal pregnancies was further demonstrated by WB (Figure S2).

### Regulation of dCD4^+^T cell function by LAG-3

**Fig. 3:**
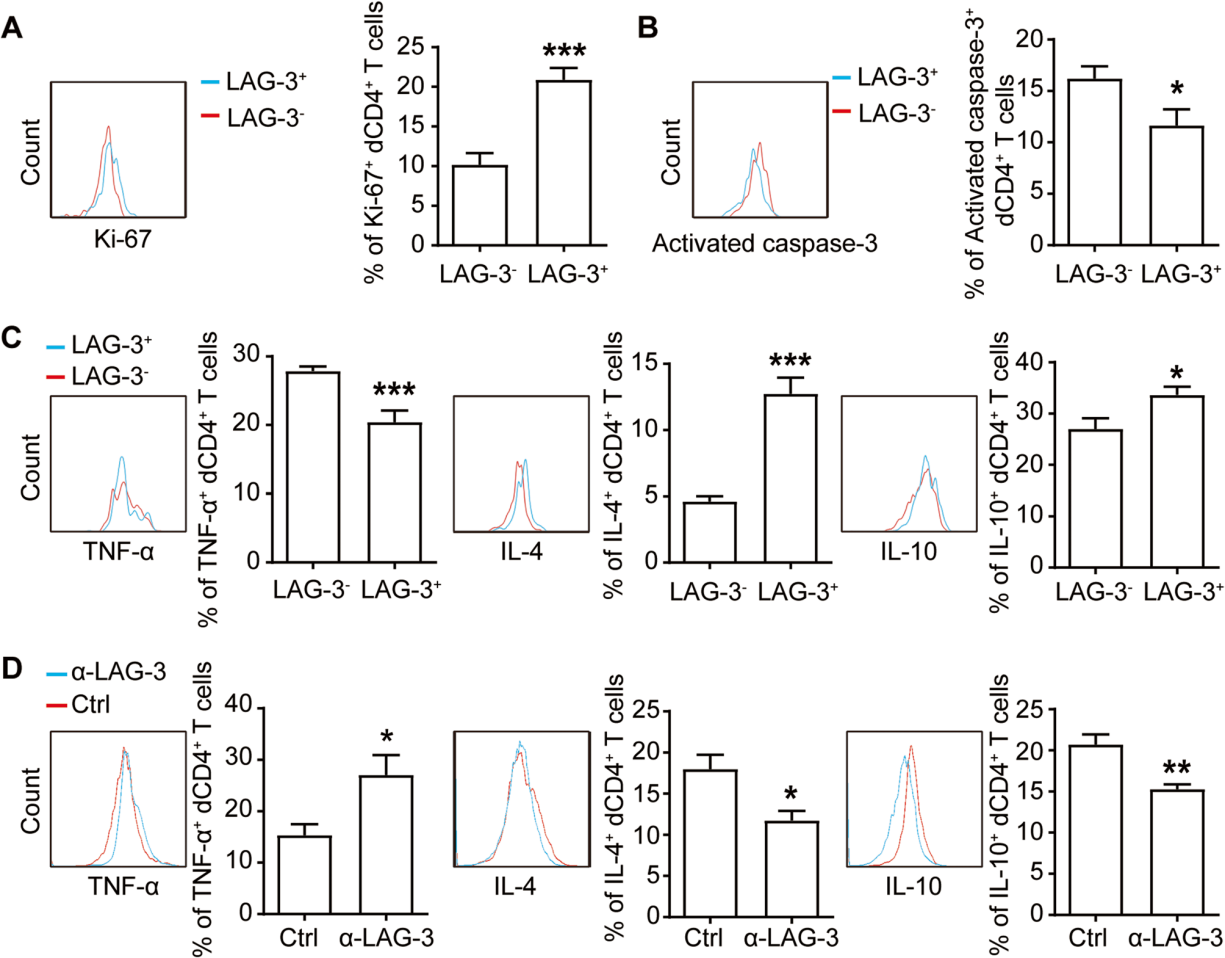
Regulatory effects of LAG-3 on dCD4^+^ T cell function.**A-C** Quantification of Ki-67 (A), activated caspase-3 (B), and TNF-α, IL-4 and IL-10 (C) staining in LAG-3^+^dCD4^+^ T cells and LAG-3^−^dCD4^+^T cells from human normal early pregnancies (*n* = 9). **D** Expression of TNF-α, IL-4 and IL-10 of dCD4^+^ T cells following a 48-hour culture with or without the addition of an anti-LAG-3 antibody (10 µg/mL). The flow cytometry plots were illustrative of three independent experiments. Data represented the mean ± SEM, *P < 0.05, **P < 0.01, ***P < 0.001

To elucidate the functional impact of LAG-3 on dCD4^+^T cells, we first examined the correlation between LAG-3 expression and dCD4^+^T cell function. Ki-67, a nuclear protein indicative of cell proliferation (Gerdes et al. 1984), was found to be significantly more abundant in LAG-3^+^dCD4^+^T cells compared to in LAG-3^−^dCD4^+^T cells (Fig. 3A). Conversely the expression of activated caspase-3 (marker of apoptosis) was lower on LAG-3^+^dCD4^+^T cells (Fig. 3B). Additionally, LAG-3^+^dCD4^+^T cells exhibited a significant upregulation in the production of IL-4 and IL-10 (Fig. 3C). These cytokines, known to be beneficial for the maintenance of pregnancy (Li et al. 2021), suggest that LAG-3 expression is associated with the proliferative capacity and the development of a tolerant phenotype of dCD4^+^T cells, potentially serving as a biomarker of healthy pregnancy.

Next, we stimulated dCD4^+^T cells with anti-CD3/CD28 antibodies, either in the presence or absence of antibodies blocking LAG-3 pathway. After a 48-hour incubation, intracellular cytokines levels in dCD4^+^T cells were analyzed. Contrary to findings in autoimmune disease and cancer(Maruhashi et al. 2022), the blockade of LAG-3 increased the production of Th1-type tumor necrosis factor (TNF)-α and interferon (IFN)-γ, but decreased Th2-type and Treg-type IL-4, IL-10 and transforming growth factor (TGF)-β1 (Fig. 3D and S3). These results implied that inhibiting LAG-3 signaling might be detrimental to the establishment and maintenance of maternal-fetal tolerance.

### LAG-3 regulated immune responses in dCD4^+^T cells so as to play important role in the maintenance of normal pregnancy

**Fig. 4:**
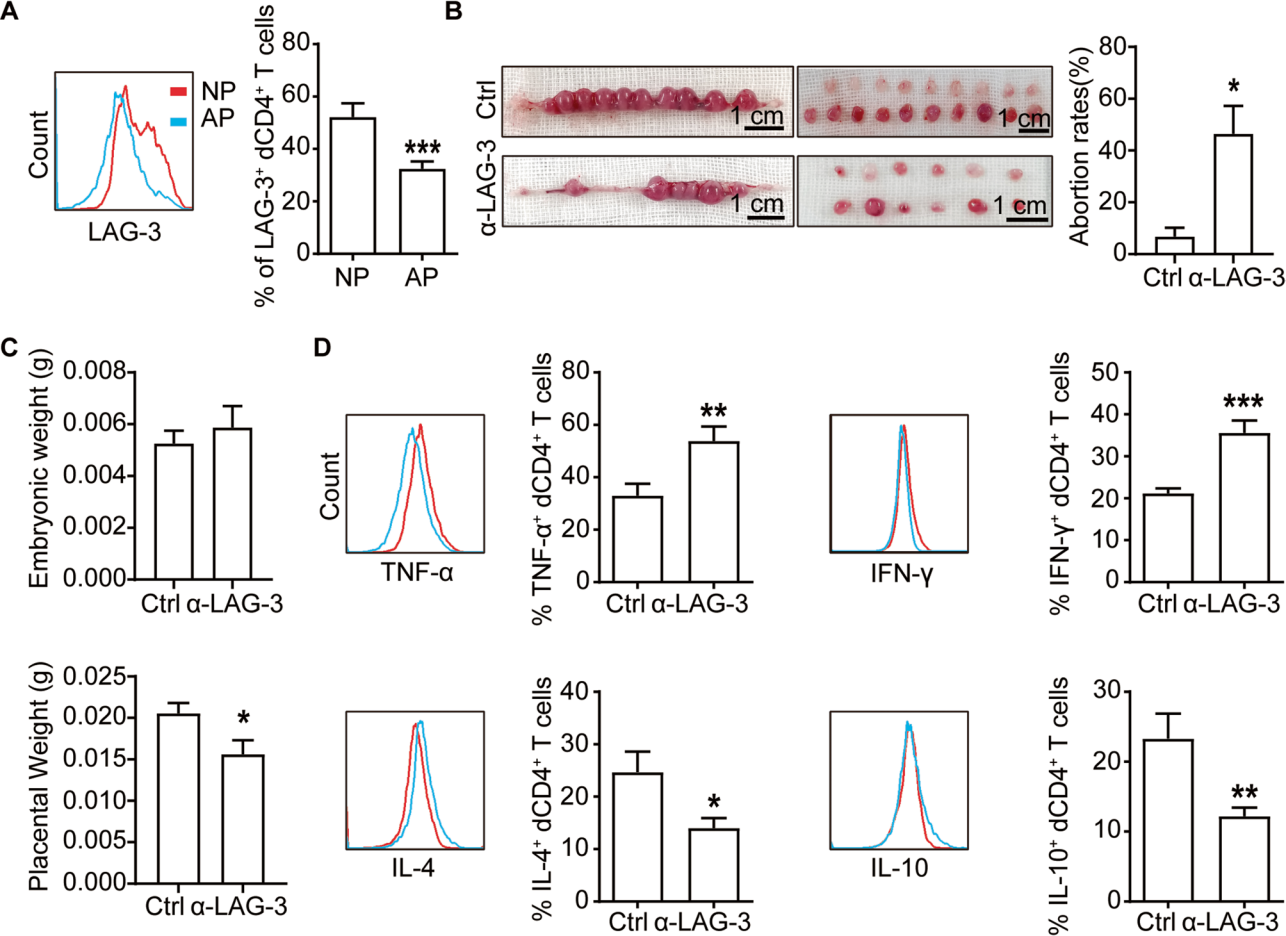
LAG-3 regulated immune responses in dCD4^+^ T cells so as to play important role in the maintenance of normal pregnancy.**A** Frequency of LAG-3^+^dCD4^+^ T cells from normal pregnancy (NP) and abortion-prone (AP) mice. **B** The percentage of fetal resorption and representative images of uterus from pregnant CBA/J females (NP) treated with either isotype IgG or anti-LAG-3 antibody. **C** Assessment of placental and fetal weights in the same treatment groups as in (B). **D** Flow cytometric quantification of cytokines production by dCD4^+^T cells from pregnant CBA/J mice following treatment with the indicated blocking antibodies. Data represented the mean ± SEM (*n* = 3–18 mice per group) and the flow cytometry plots were representative of four independent analyses. *P < 0.05, **P < 0.01, ***P < 0.001, compared to the control group

To further clarify whether functional regulation of LAG-3 on CD4^+^T cells is involved in pregnancy maintenance, we first compared LAG-3 expression on dCD4^+^T cells from mouse model representing normal pregnancy and those prone to abortion. We used a well-established allogeneic pregnancy model that involves mating female CBA/J mice with male BALB/c mice, and crossed female CBA/J with male DBA/2 mice to create an abortion-prone model. Our findings mirrored those observed in human dCD4^+^T cells, with a notable reduction in LAG-3^+^dCD4^+^ T cells within the abortion-prone mouse model (Fig. 4A).

In the second assay, we assessed the impact of a LAG-3-blocking antibody on normal pregnant CBA/J females (female CBA/J × male BALB/c mice). The administration of this blocking antibody caused an increased rate of embryo resorption (Fig. 4B), and a significant decrease in the placental weight, but had no effect on fetal weight (Fig. 4C). These data indicated that LAG-3-blocking antibody may exert adverse effects on murine fertility.

To explore whether the increased fetal loss in treated mice could be attributed to the compromised function of dCD4^+^T cells under anti-LAG-3 antibody treatment, we analyzed dCD4^+^T cells from the treated mice. Our analysis revealed a decrease in the production of IL-4 and IL-10, alongside an increase in TNF-α and IFN-γ production (Fig. 4D). These *in vivo* results, in conjunction with our *in vitro* data, supported the notion that LAG-3 played a crucial role in modulating immune responses in dCD4^+^T cells, thereby contributing significantly to the maintenance of normal pregnancy.

### Palmitoylation involved in regulating LAG-3 expression during RPL

**Fig. 5:**
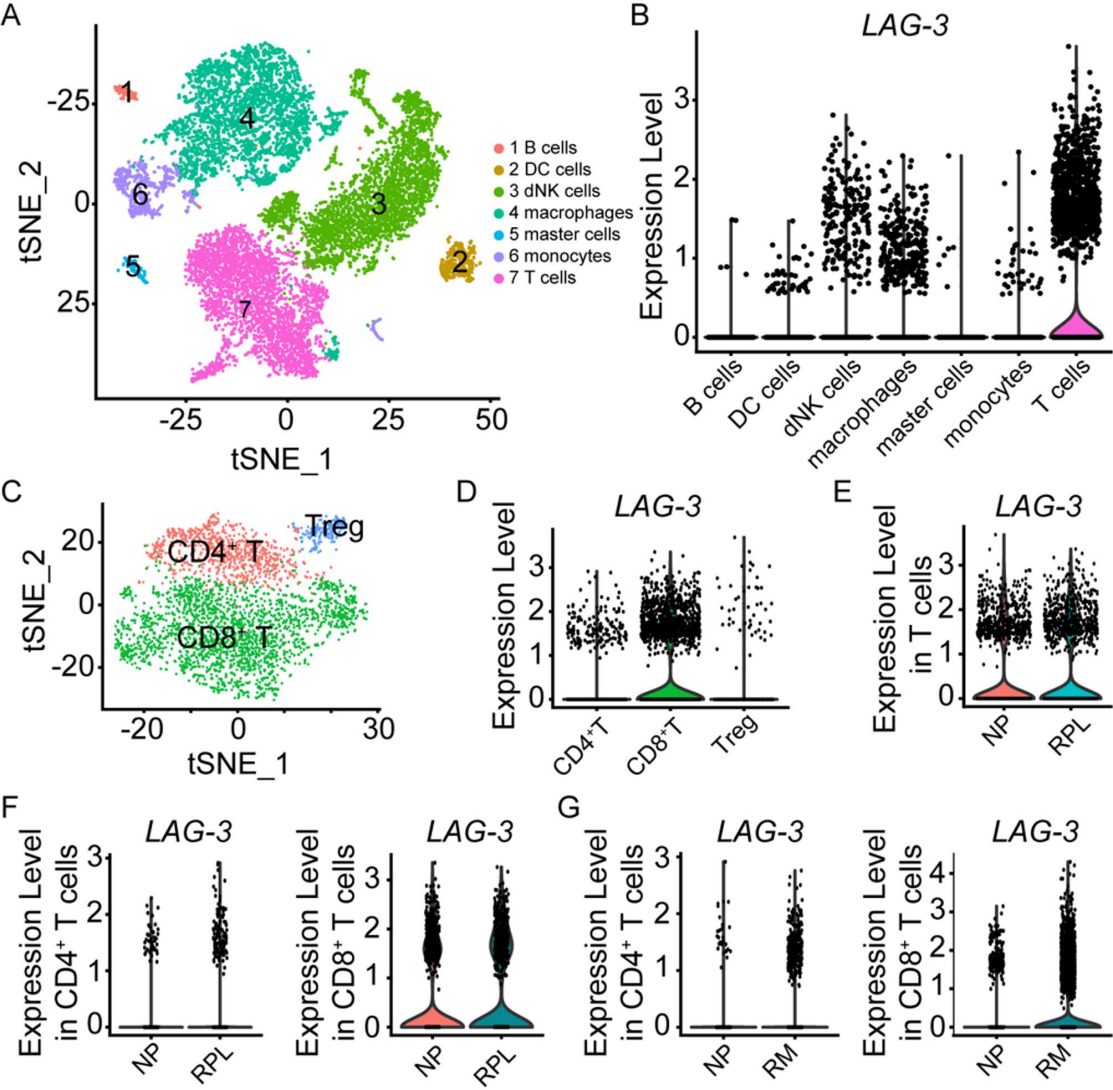
Expression profiles of LAG-3 of decidual immune cells between the clinically normal first trimester pregnancies and RPL patients from single-cell databases.**A** TSNE plot derived from single-cell RNA sequencing (scRNA-seq) data, illustrating the distribution of seven distinct cell types in decidual tissues during early pregnancy. **B** Violin plots depicting the expression of *LAG-3* in the indicated cells. **C** TSNE plot of the T cells as defined in (A), with each cell color-coded to represent its associated cell type. **D** Violin plots showing the expression of *LAG-3* in the indicated subpopulations. **E**,** F ***LAG-3* expression of T cells (E), CD4^+^T cells and CD8^+^T cells (F) in normal pregnancy (NP) and RPL patients (RPL). **G** Violin plots displaying the *LAG-3* expression in the CD4^+^T cells and CD8^+^T cells in NP and recurrent miscarriages (RM) from another independent single-cell transcriptomic profiling of decidual immune cells

To elucidate the role of LAG-3 in early pregnancy, we conducted an analysis of two published single-cell databases(Chen et al. 2021; Wang et al. 2021). Firstly, we re-analyzed the single-cell transcriptomic profiling of decidual immune cells obtained from three normal pregnancies and three RPL patients(Chen et al. 2021). Through unsupervised graph-based clustering of the integrated dataset, we identified seven distinct cell types (Fig. 5A and S4A-4B). Notably, decidual T cells exhibited a substantially higher expression of *LAG-3* than other decidual immune cells (Fig. 5B). We further clustered the decidual T cells into three subsets based on their gene expression signatures, including CD4^+^T cells, CD8^+^T cells, and Tregs (Fig. 5C and S4C). All three subsets of decidual T cells demonstrated certain expression of *LAG-3* (Fig. 5D). Contrary to our expectations, *LAG-3* expression on the total decidual T cells (Fig. 5E), as well as on dCD4^+^T cells and dCD8^+^T cells (Fig. 5F) were found to be similar between normal pregnancies and RPL cases, which was diverged from the findings presented in our Fig. 2. Similar phenomenon was observed in an independent single-cell transcriptomic profiling of decidual immune cells from normal pregnancies and RPL patients(Wang et al. 2021), where *LAG-3* expression on dCD4^+^T cells and dCD8^+^T cells had no difference between the two groups (Fig. 5G). These results suggested that while the protein level of LAG-3 on dCD4^+^T cells decreased in RPL, the RNA level of *LAG-3* remained stable in RPL, pointing to a potential role for posttranslational modification(Y. F. Wang and Tong 2023) in the regulation of LAG-3 protein levels during RPL.

**Fig. 6:**
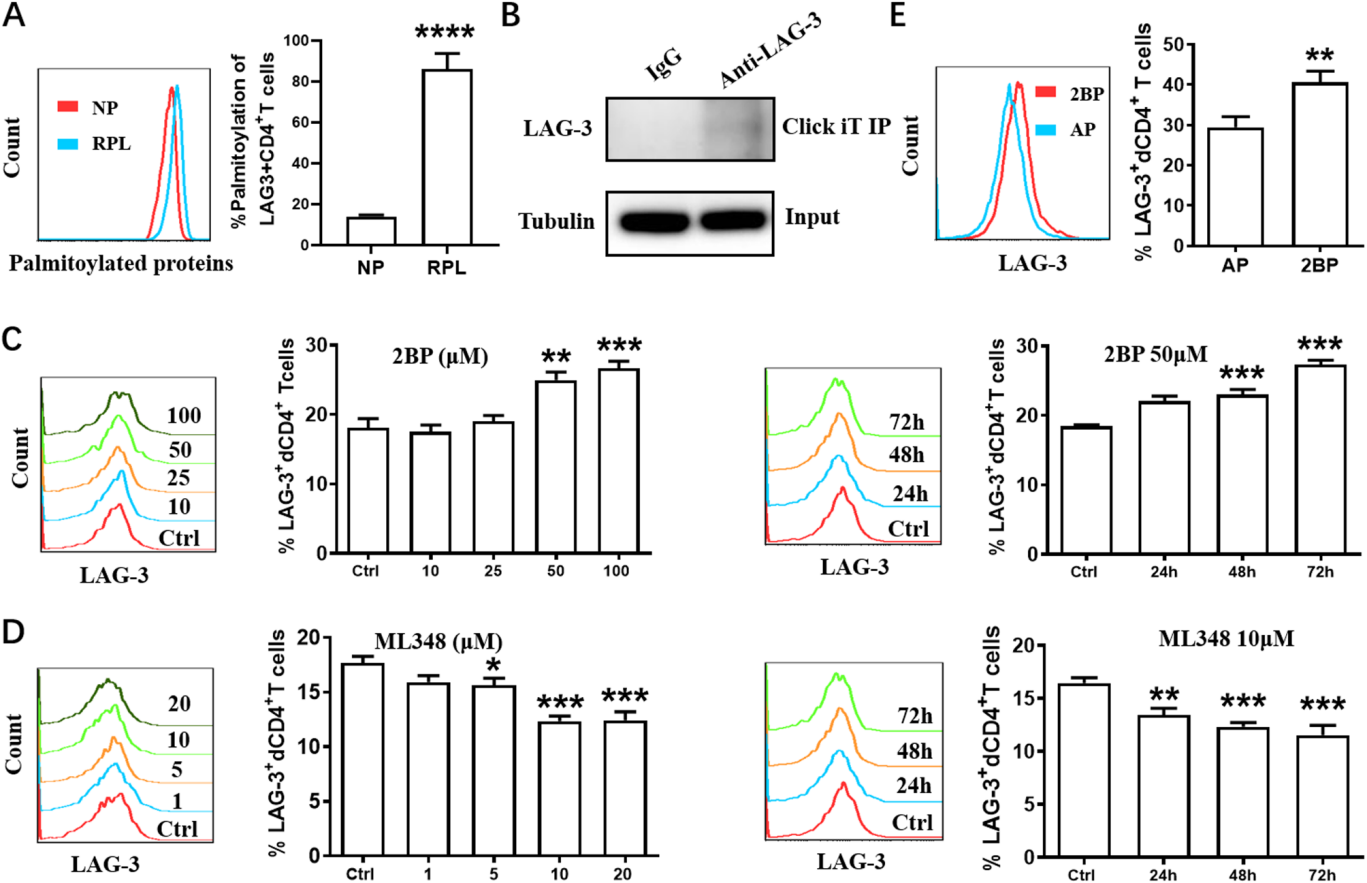
Palmitoylation participated in regulating LAG-3 expression during RPL.**A** Flow cytometric analysis (left) and quantification (right) of palmitoylation of LAG-3^+^CD4^+^T cells. **B** Cells were cultured in medium containing palmitic acid-azide and the cell lysates were prepared for the Click-iT reaction, followed by immunoblotting with the LAG-3 antibody. IP, immunoprecipitation. **C** Flow cytometric analysis and quantification of LAG-3 expression on dCD4^+^T cells treated with 2BP at the indicated concentrations for 48 h, or with 50 µM 2BP for 24–72 h. **D** Flow cytometric analysis and quantification of LAG-3 expression on dCD4^+^T cells treated with ML348 at the indicated concentrations for 48 h, or with 10 µM ML348 for 24–72 h. **E** Frequency of LAG-3^+^dCD4^+^ T cells from AP mice treated with or without 2BP. Data represent mean ± SEM. *P < 0.05, **P < 0.01, ***P < 0.001, ****P < 0.0001

Our palmitoylation proteomics analysis provided further insights, revealing increased palmitoylation of LAG-3 in RPL compared to normal pregnancy (data not shown). The palmitoylation of LAG-3^+^dCD4^+^T cells was found to be elevated in RPL (Fig. 6A), and LAG-3 palmitoylation was verified by click-iT chemistry (Fig. 6B). Furthermore, the general palmitoylation inhibitor, 2-bromopalmitate (2BP), upregulated LAG-3 expression on dCD4^+^T cells both in dose-dependent and time-dependent manners (Fig. 6C). In contrast, enhanced palmitoylation by selective inhibitors of acyl protein thioesterase 1, ML348, decreased the expression of LAG-3 on dCD4^+^T cells both in dose-dependent and time-dependent manners (Fig. 6D). Consistent with these observations, 2BP also increased LAG-3 expression on dCD4^+^T cells from abortion-prone mouse model. These findings suggested that palmitoylation might participate in regulating LAG-3 expression during RPL.

## Discussion

Pregnancy is a fascinating and complex biological process that challenges traditional immunological paradigms. The mechanisms that safeguard the allogeneic fetal tissues from maternal immune rejection are numerous and multifaceted, yet our understanding of these protective processes remains elementary. Understanding the immunological shifts that occur during pregnancy becomes crucial for demystifying the maternal-fetal interface. These immunological dynamics are pivotal for the maintenance of pregnancy and could potentially inform innovative strategies for improving pregnancy outcomes, with broader implications for fields such as transplantation and autoimmunity, in which long-lasting antigen-specific tolerance is sought. In the present study, we presented evidence that LAG-3 was a key regulator of dCD4^+^T cell function and therefore played important roles in the maintenance of normal pregnancy.

LAG-3, an immune checkpoint receptor of the immunoglobulin superfamily, has been implicated in self-tolerance, as evidenced by studies showing reduced LAG-3^+^CD4^+^ T cells in patients with active psoriatic arthritis(Gertel et al. 2021). Furthermore, serum LAG-3 represents a promising diagnostic biomarkers for detecting the RPL with prethrombotic state(Wu et al. 2021). Our research indicates that LAG-3 expression in dCD4^+^T cells was notably diminished in RPL and acted as a modulator of RPL. The increased expression of LAG-3 on dCD4^+^T cells during pregnancy might be attributed to local microenvironment at the maternal-fetal interface, with embryonic trophoblasts contributing to the elevated LAG-3 expression on dCD4^+^T cells.

There is a general consensus that LAG-3 contributes to the regulation of activated T cell expansion(Angin et al. 2020; Maçon-Lemaître and Triebel 2005). Early research posited LAG-3 as a negative regulator of T cell activation and function, with blocking LAG-3 leading to enhanced proliferation and Th1 cytokine production of CD4^+^T cells(Huard et al. 1994). In contrast, we found that LAG-3^+^dCD4^+^T cells expressed higher Ki-67 and produced a greater array of anti-inflammatory or regulatory cytokines, thereby promoting maternal-fetal tolerance. Our investigations into the functionality of LAG-3 on dCD4^+^T cells in RPL subjects and the consequences of reduced LAG-3 expression demonstrated that LAG-3 blockade resulted in decreased production of anti-inflammatory cytokines of dCD4^+^T cells, both* in vivo* and *in vitro.*

Clinical trials of LAG-3-blocking agents have developed rapidly(Li et al. 2023). However, our study showed that pregnant CBA/J females treated with LAG-3 blocking antibodies became more susceptible to fetal loss, indicating that the reduction proportions and the functional abnormity of LAG-3 might be one of the contributing factors to miscarriage. Since the immune checkpoint pathways are critical in self-tolerance and immunological homeostasis, and their role in pregnancy is important for protecting the fetus from the maternal immune responses(Garutti et al. 2021). Blockage of these immune checkpoints could result in fetal rejection by the maternal responses(Zhang et al. 2015). Additionally, immune checkpoint inhibitors have been reported to cross the placental and increase the risk of fetal disorders(Garutti et al. 2021), suggesting that their use during pregnancy are not recommended(Noseda et al. 2023). However, the use of these inhibitors before pregnancy, or after conception, when most babies are born healthy with no fetal abnormalities, has been reported as beneficial(Andrikopoulou et al. 2021; Gambichler and Susok 2022; Le-Nguyen et al. 2022). Nevertheless, other studies have linked the use of immune checkpoint inhibitors to premature deliveries, fetal distress syndrome, spontaneous abortions, and intrauterine growth restriction(Andrikopoulou et al. 2021), underscoring the need for caution regarding reproductive safety when employing immune checkpoint inhibitors.

Our data reveal a striking finding: while the protein level of LAG-3 on dCD4^+^T cells decreased in RPL, compared to normal pregnancy, the RNA level of *LAG-3*, as analyzed from the two published single-cell databases, remained stable. We hypothesize that palmitoylation, a post-translational lipid modification, might participate in regulating LAG-3 expression During RPL. Palmitoylation, which involves the attachment of the 16-carbon fatty acid palmitate to proteins, is known to regulate protein stability, localization and function(Chen et al. 2024). For example, inhibiting the palmitoylation of programmed-death Ligand 1 has been shown to decrease its expression and enhanced T-cell immune responses against tumors(Yao et al. 2019). Our findings indicated that palmitoylation of LAG-3^+^dCD4^+^ T cells was increased in RPL, and the general palmitoylation inhibitor 2BP upregulated LAG-3 expression on dCD4^+^T cells both *in vitro* and *in vivo*. In contrast, enhanced palmitoylation induced by ML348 reduced the expression of LAG-3 on dCD4^+^T cells. High-fat diet, which could promote palmitoylation(Bu et al. 2024) have been linked to adverse pregnancy outcomes(Yang et al. 2024), suggesting a potential link between diet factors, immune checkpoint expression and pregnancy outcomes, though still warrants further investigation.

## Conclusion

**Fig. 7:**
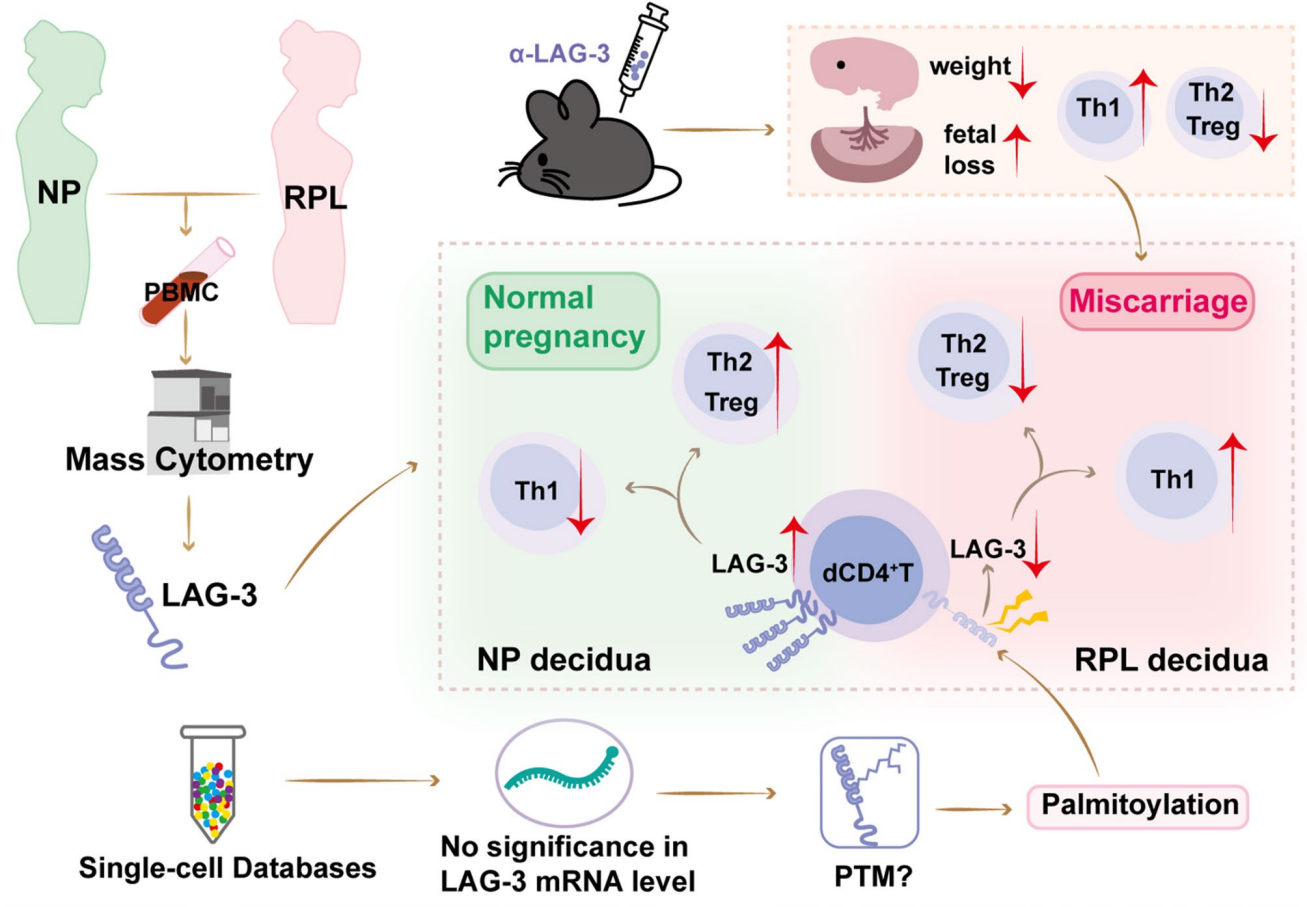
Schematic diagram of functional regulation of LAG-3 on CD4^+^ T cells at the maternal-fetal interface. CyTOF data showed higher LAG-3 expression during normal pregnancy. LAG-3^+^dCD4^+^T cells are involved in the production of anti-inflammatory or regulatory cytokines. Blockade LAG-3 pathways can disrupt the normal function of dCD4^+^T cells, leading to an increased risk of fetal loss. Decreased LAG-3 expression on dCD4^+^T cells might be associated with miscarriage due to the impaired functionality of dCD4^+^T cells. However, the scRNA-seq data showed that RNA levels of LAG-3 on dCD4^+^T cells were similar between NP and RPL. This suggests that post-translational modifications, such as palmitoylation, might be involved in the downregulation of LAG-3 expression, which is also associated with RPL

In conclusion (Fig. 7), our study concluded the higher LAG-3 expression on dCD4^+^T cells during normal pregnancy is beneficial, and decreased LAG-3 on dCD4^+^T cells might be associated with miscarriage. LAG-3^+^dCD4^+^T cells produced more anti-inflammatory cytokines. Palmitoylation was associated with lower LAG-3 expression during RPL. Blockade of LAG-3 led to dCD4^+^T cells dysfunction and increased fetal loss. While LAG-3 might represent promising early warming targets of RPL, further research is needed to elucidate the downstream signaling mechanisms of LAG-3. A deeper understanding of the LAG-3 modulation pathway could lead to more efficacious therapies and the identification of biomarkers for the diagnosis and treatment of RPL.

## Supplementary Information

Supplementary Material 1: Fig S1. Comparative LAG-3 expression profiles between the clinically normal first trimester pregnancies and RPL patients. A TSNE plot of PBMCs from NP and RPL. B Heatmap shows mean expression values of indicated proteins, normalized per column by z-score. C, D Normalized LAG-3 expression of the entire PBMCs from NP and RPL. Fig S2. The protein level of LAG-3 in dCD4^+^T cells from NP and RPL examined by western blot. Images are representative of three individual experiments. Fig S3. Expression of IFN-γ and TGF-β1 of dCD4^+^ T cells cultured for 48 h in the presence or absence of anti-LAG-3 antibody (10 μg/mL). The flow cytometry plots were representative of three independent experiments. Data represented the mean ±SEM, *P<0.05,***P<0.001. Fig S4. A Clustree showing cell clustering at various resolutions. B UMAP plot (Left) showing populations of PBMCs from NP and RPL and violin plots (Right) of the normalized *LAG3* expression in PBMCs from NP and RPL. C UMAP plot (Left) showing T cell populations from NP and RPL and violin plots (Right)representing the normalized LAG3 expression in the indicated T cell subsets from NP and RPL. Supplementary Table 1. Clinical characteristics of enrolled subjects. Supplementary Table 2. Antibody list.

## Data Availability

No datasets were generated or analysed during the current study.

## Abbreviations

RPL: Recurrent pregnancy loss
LAG-3: Lymphocyte activation gene-3
dCD4^+^T: Decidual CD4^+^T
Treg: Regulatory T cell
PD-1: Programmed cell death protein 1
CTLA-4: Cytotoxic T-lymphocyte antigen-4
TIM-3: T cell immunoglobulin and mucin domain containing-3
CyTOF: Cytometry by time-of-flight
NP: Normal pregnancies
PBMCs: Peripheral blood mononuclear cells
PBS: Phosphate buffer solution
PMA: Phorbol 12-myrstate 13-acetate
2BP: 2-Bromopalmitate
FBS: Fetal bovine serum
GEO: Gene Expression Omnibus
HB: Hemoglobin
SEM: Standard error of mean
ANOVA: One-way analysis of variance
pCD4^+^T: peripheral CD4^+^T
pCD8^+^T: peripheral CD8^+^T
TNF: Tumor necrosis factor
IFN: Interferon
TGF: Transforming growth factor
