# Supplementary material for: LAG-3 palmitoylation-inducing dysfunction of decidual CD4+T cells is associated with recurrent pregnancy loss
Source: Mol Med. 2025 Sep 29;31:298. doi: 10.1186/s10020-025-01361-9 (PMC12482660; doi:10.1186/s10020-025-01361-9)
Supplement: Supplementary file 1 — Supplementary Material 1: Fig S1. Comparative LAG-3 expression profiles between the clinically normal first trimester pregnancies and RPL patients. A TSNE plot of PBMCs from NP and RPL. B Heatmap shows mean expression values of indicated proteins, normalized per column by z-score. C, D Normalized LAG-3 expression of the entire PBMCs from NP and RPL. Fig S2. The protein level of LAG-3 in dCD4+T cells from NP and RPL examined by western blot. Images are representative of three individual experiments. Fig S3. Expression of IFN-γ and TGF-β1 of dCD4+ T cells cultured for 48 h in the presence or absence of anti-LAG-3 antibody (10 μg/mL). The flow cytometry plots were representative of three independent experiments. Data represented the mean ±SEM, *P<0.05,***P<0.001. Fig S4. A Clustree showing cell clustering at various resolutions. B UMAP plot (Left) showing populations of PBMCs from NP and RPL and violin plots (Right) of the normalized LAG3 expression in PBMCs from NP and RPL. C UMAP plot (Left) showing T cell populations from NP and RPL and violin plots (Right)representing the normalized LAG3 expression in the indicated T cell subsets from NP and RPL. Supplementary Table 1. Clinical characteristics of enrolled subjects. Supplementary Table 2. Antibody list. [file 10020_2025_1361_MOESM1_ESM.docx]

**Supplementary Materials**


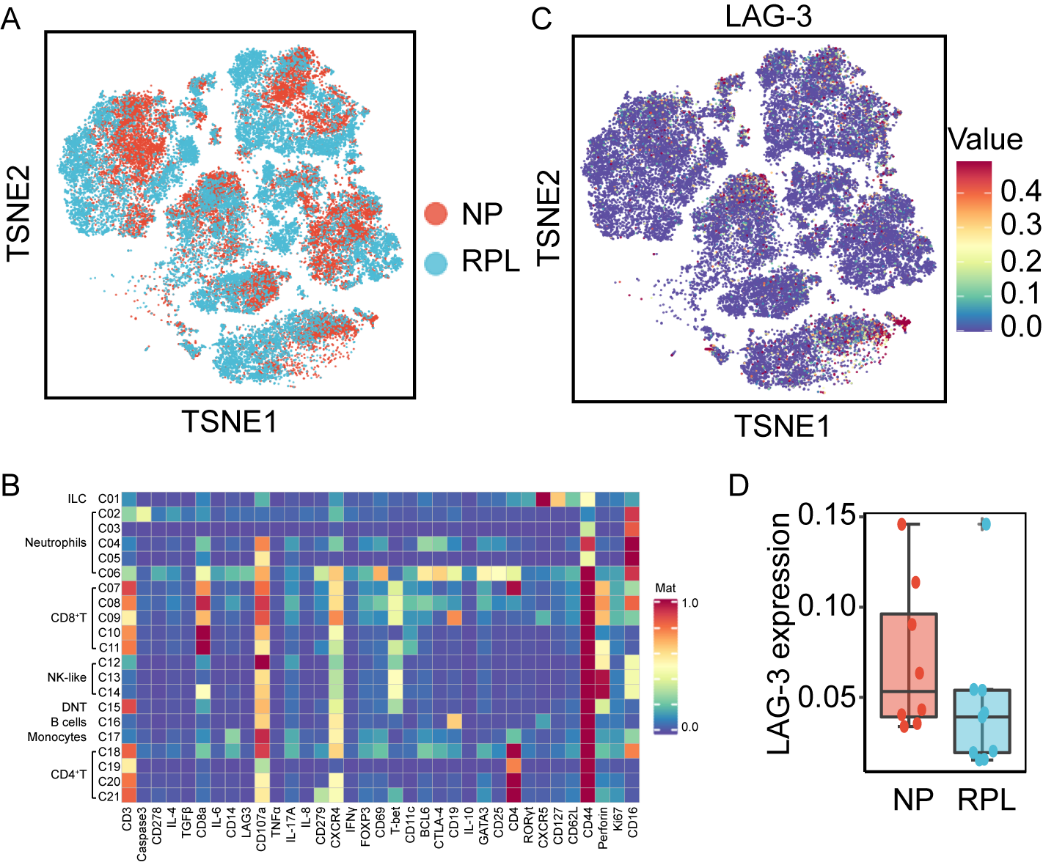


**Fig.S1 Comparative LAG-3 expression profiles between the clinically normal first trimester pregnancies and RPL patients.**

A TSNE plot of PBMCs from NP and RPL. B Heatmap shows mean expression values of indicated proteins, normalized per column by z-score. C, D Normalized LAG-3 expression of the entire PBMCs from NP and RPL.


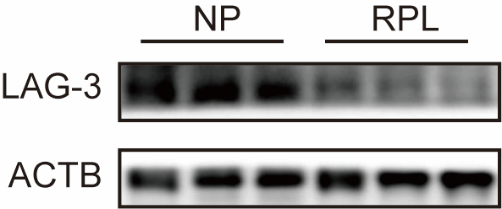


**Fig.S2** The protein level of LAG-3 in dCD4^+^T cells from NP and RPL examined by western blot. Images are representative of three individual experiments.


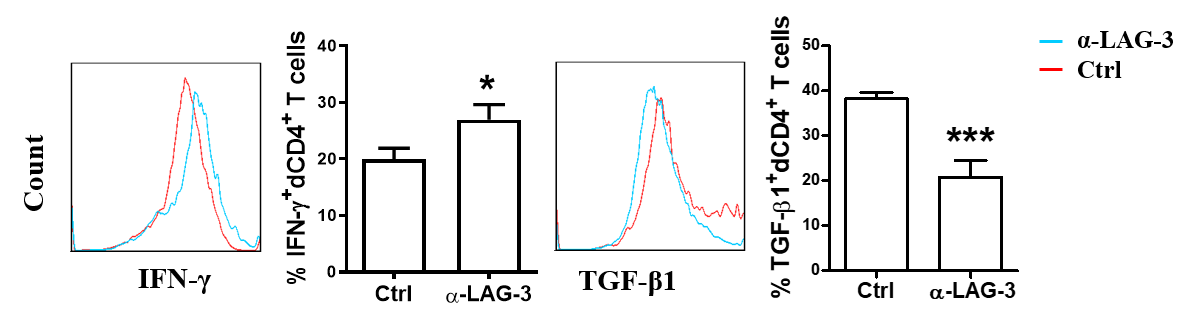


**Fig.S3** Expression of IFN-γ and TGF-β1 of dCD4^+^ T cells cultured for 48 h in the presence or absence of anti-LAG-3 antibody (10 μg/mL). The flow cytometry plots were representative of three independent experiments. Data represented the mean ±SEM, *P<0.05, ***P<0.001.


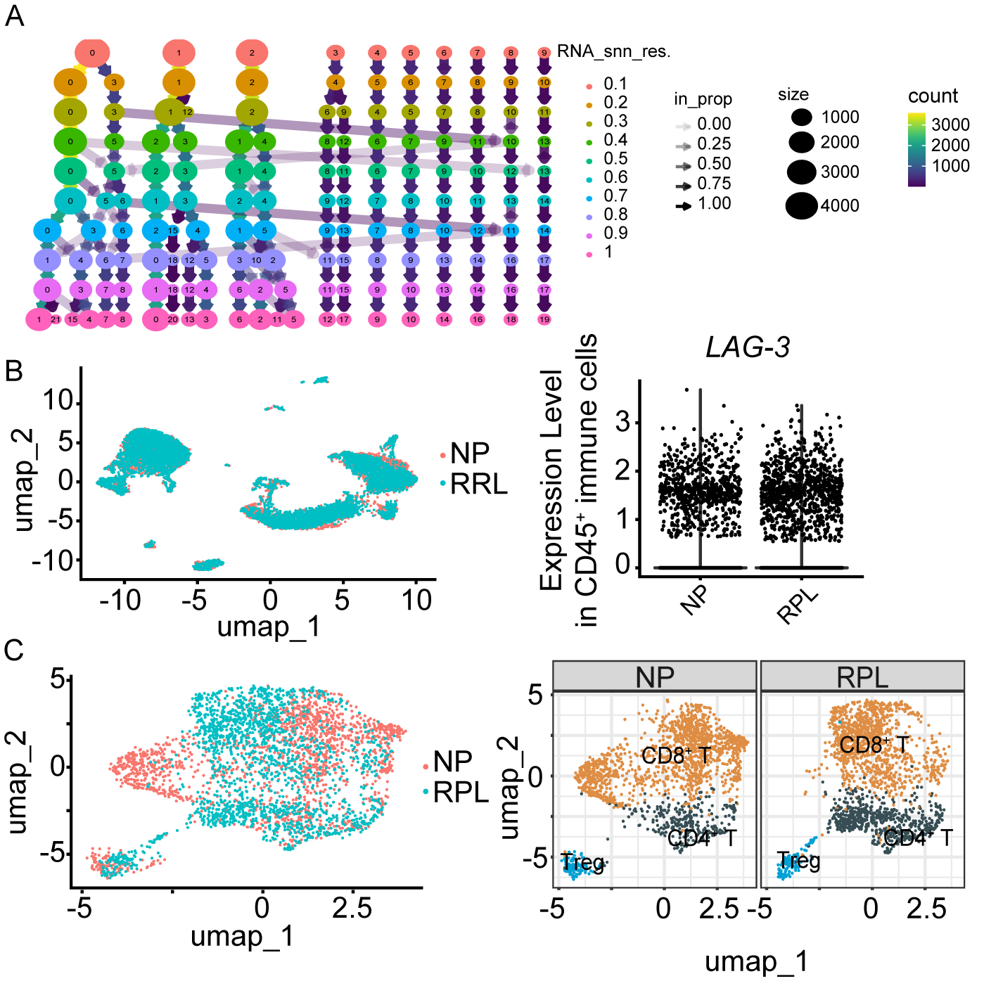


**Figure S4.** (A) Clustree showing cell clustering at various resolutions. (B) UMAP plot (Left) showing populations of PBMCs from NP and RPL and violin plots (Right) of the normalized *LAG3* expression in PBMCs from NP and RPL. (C) UMAP plot (Left) showing T cell populations from NP and RPL and violin plots (Right)representing the normalized LAG3 expression in the indicated T cell subsets from NP and RPL.

**Supplementary Table 1 Clinical characteristics of enrolled subjects.**

| Subjects^b^ | NP (n=55) | RPL (n=26) | P-value |
| --- | --- | --- | --- |
| Age mean (years)^a^ | 29.96±0.60 | 30.50±0.95 | 0.62 |
| Age range (years) | 20-41 | 23-38 | **-** |
| Previous pregnancy loss^a^ | 0 | 2.426±0.28 | <0.0001 |
| Previous normal births^a^ | 1.527±0.09 | 0 | <0.0001 |
| Pregnancy week (sample collected)^a^ | 7.332±0.12 | 7.230±0.17 | 0.63 |

a Values are expressed as the median ± SEM. RPL was defined as spontaneous abortion in patients who also had a history of two or more consecutive pregnancy loss without known causes.

b None of the subjects had any history of treatment.

Groups: NP, normal pregnancy; RPL, recurrent pregnancy loss.

**Supplementary Table 2 Antibody list.**

| Cat# | Antigen | Vendor | Mass Tag | Vendor |
| --- | --- | --- | --- | --- |
| 300443 | Purified anti-human CD3 (MaxPar® Ready) | Biolegend | 141Pr | Fluidigm |
| 300541 | Purified anti-human CD4 (MaxPar® Ready) | Biolegend | 169Tm | Fluidigm |
| 301053 | Purified anti-human CD8a (MaxPar® Ready) | Biolegend | 146Nd | Fluidigm |
| 301639 | Purified anti-human CD11c (MaxPar® Ready) | Biolegend | 162Dy | Fluidigm |
| 301843 | Purified anti-human CD14 (MaxPar® Ready) | Biolegend | 148Nd | Fluidigm |
| 302051 | Purified anti-human CD16 (MaxPar® Ready) | Biolegend | 209Bi | Fluidigm |
| 302247 | Purified anti-human CD19 (MaxPar® Ready) | Biolegend | 165Ho | Fluidigm |
| 306523 | Purified anti-human CD184 (CXCR4) (MaxPar® Ready) | Biolegend | 156Gd | Fluidigm |
| 310939 | Purified anti-human CD69 (MaxPar® Ready) | Biolegend | 160Gd | Fluidigm |
| 338811 | Purified anti-human CD44 (MaxPar® Ready) | Biolegend | 174Yb | Fluidigm |
| 318302 | Purified anti-human CD56 (MaxPar® Ready) | Biolegend | 149Sm | Fluidigm |
| 350523 | Purified anti-human Ki-67 (MaxPar® Ready) | Biolegend | 176Yb | Fluidigm |
| 500829 | Purified anti-human IL-4 (MaxPar® Ready) | Biolegend | 144Nd | Fluidigm |
| 501115 | Purified anti-human IL-6 (MaxPar® Ready) | Biolegend | 147Sm | Fluidigm |
| 502941 | Purified anti-human TNF-α (MaxPar® Ready) | Biolegend | 152Sm | Fluidigm |
| 506521 | Purified anti-human IFN-γ (MaxPar® Ready) | Biolegend | 158Gd | Fluidigm |
| 328635 | Purified anti-human CD107a (LAMP-1) (MaxPar® Ready) | Biolegend | 151Eu | Fluidigm |
| 351337 | Purified anti-human CD127 (IL-7Rα) (MaxPar® Ready) | Biolegend | 172Yb | Fluidigm |
| 329941 | Purified anti-human CD279 (PD-1) (MaxPar® Ready) | Biolegend | 155Gd | Fluidigm |
| 304835 | Purified anti-human CD62L (MaxPar® Ready) | Biolegend | 173Yb | Fluidigm |
| 501423 | Purified anti-human IL-10 (MaxPar® Ready) | Biolegend | 166Er | Fluidigm |
| 512331 | Purified anti-human IL-17A (MaxPar® Ready) | Biolegend | 153Eu | Fluidigm |
| 644825 | Purified anti-T-bet (MaxPar® Ready) | Biolegend | 161Dy | Fluidigm |
| 302602 | Purified anti-human CD25 | Biolegend | 168Er | Fluidigm |
| 356902 | Purified anti-human CD185 (CXCR5) | Biolegend | 171Yb | Fluidigm |
| 349902 | Purified anti-human CD152 (CTLA-4) | Biolegend | 164Dy | Fluidigm |
| 349602 | Purified anti-human LAP (TGF-β1) | Biolegend | 145Nd | Fluidigm |
| 308102 | Purified anti-human Perforin | Biolegend | 175Lu | Fluidigm |
| 653802 | Purified anti-GATA3 Antibody | Biolegend | 167Er | Fluidigm |
| 369202 | Purified anti-human CD223 (LAG-3) | Biolegend | 150Nd | Fluidigm |
| MAB10753 | Anti-Caspase3 (active form), clone 3D9.3 | Merck | 142Nd | Fluidigm |
| 313502 | Purified anti-human/mouse/rat CD278 (ICOS) | Biolegend | 143Nd | Fluidigm |
| 514602 | Purified anti-human IL-8 | Biolegend | 154Sm | Fluidigm |
| 320102 | Purified anti-human FOXP3 | Biolegend | 159Tb | Fluidigm |
| 562197 | Purified Mouse Anti-RORγt | BD | 170Er | Fluidigm |
| 648302 | Purified anti-mouse/human Bcl-6 | Biolegend | 163Dy | Fluidigm |
| 201192A | Intercalator | Fluidigm | 193Ir | Fluidigm |
| 1134357 | Cisplatin | Merck | 195Pt | Fluidigm |
